# Geographic mode of speciation in a mountain specialist Avian family endemic to the Palearctic

**DOI:** 10.1002/ece3.539

**Published:** 2013-04-18

**Authors:** Sergei V Drovetski, Georgy Semenov, Sofya S Drovetskaya, Igor V Fadeev, Yaroslav A Red'kin, Gary Voelker

**Affiliations:** 1 Tromsø University MuseumNO-9037, Tromsø, Norway; 2 Institute of Systematics and Ecology of Animals of Siberian Branch of Russian Academy of SciencesFrunze St. 11, 630091, Novosobirsk, Russia; 3 19355 53rd Ave. NE, Lake Forest Park, WA, 98155; 4 State Darwin MuseumVavilova St. 57, 117292, Moscow, Russia; 5 Zoological Museum of Moscow State UniversityBol'shaya Nikitskaya St. 6, 103009, Moscow, Russia; 6 Department of Wildlife and Fisheries Sciences, Biodiversity Research and Teaching Collections, Texas A&M UniversityCollege Station, TX, 77843

**Keywords:** Allopatric speciation, Himalayas, historical biogeography, Palearctic, phylogeny, Prunellidae

## Abstract

Mountains host greater avian diversity than lowlands at the same latitude due to their greater diversity of habitats stratified along an elevation gradient. Here we test whether this greater ecological heterogeneity promotes sympatric speciation. We selected accentors (Prunellidae), an avian family associated with mountains of the Palearctic, as a model system. Accentors differ in their habitat/elevation preferences and south-central Siberia and Himalayan regions each host 6 of the 13 species in the family. We used sequences of the mtDNA ND2 gene and the intron 9 of the Z chromosome specific ACO1 gene to reconstruct a complete species-level phylogeny of Prunellidae. The tree based on joint analysis of both loci was used to reconstruct the family's biogeographic history and to date the diversification events. We also analyzed the relationship between the node age and sympatry, to determine the geographic mode of speciation in Prunellidae. Our data suggest a Miocene origin of Prunellidae in the Himalayan region. The major division between alpine species (subgenus *Laiscopus*) and species associated with shrubs (subgenus *Prunella*) and initial diversification events within the latter happened within the Himalayan region in the Miocene and Pliocene. Accentors colonized other parts of the Palearctic during the Pliocene-Pleistocene transition. This spread across the Palearctic resulted in rapid diversification of accentors. With only a single exception dating to 0.91 Ma, lineages younger than 1.5 Ma are allopatric. In contrast, sympatry values for older nodes are >0. There was no relationship between node age and range symmetry. Allopatric speciation (not to include peripatric) is the predominant geographic mode of speciation in Prunellidae despite the favorable conditions for ecological diversification in the mountains and range overlaps among species.

## Introduction

Allopatric speciation appears to be the dominant geographic mode of speciation in birds (Mayr 1942; Cracraft 1982; Chesser and Zink 1994; Friesen and Anderson 1997; Barraclough and Vogler 2000; Coyne and Price 2000; Drovetski 2003; Phillimore et al. 2008; Price 2008). Sympatric speciation is infrequent and generally associated with unusual reproductive circumstances. For example, African indigobirds (*Vidua* spp.) that parasitize nests of estrildid finches (Estrildidae) could have diverged sympatrically through host specialization (Sorenson et al. 2003). But overall, nest parasitism is uncommon across avian lineages, with host-specificity in parasitism and, therefore, the possibility for sympatric speciation being even less common. In the band-rumped storm-petrel (*Oceanodroma castro*), populations on several Atlantic archipelagos diverge due to breeding allochrony with birds breeding in different seasons in the same archipelago being evolutionary sister-units (Friesen et al. 2007). However, allochrony is not common in birds, even among other storm-petrels.

The intriguing question then is what conditions (if any) can facilitate sympatric speciation in birds? Adaptive radiation appears to be a likely scenario for sympatric speciation. However, studies of avian adaptive radiations on oceanic islands provide virtually no support for sympatric speciation (Coyne and Price 2000). In the Hawaiian honeycreepers (Drepanidinae), only 2 of 7 sister pairs occur on the same island – the Nihoa (*Telespiza ultima*) and Laysan (*T. cantans*) finches on Laysan, and the apapane (*Himatione sanguinea*) and akohekohe (*Palmeria dolei*) on Maui (Lerner et al. 2011). In both pairs, one species has a much wider distribution in the archipelago than the other, so the potential for allopatric speciation cannot be excluded. In the Galapagos Islands, all mockingbird lineages are allopatric (Arbogast et al. 2006) and for Darwin's finches, allopatry is proposed as the initial stage of speciation (Petren et al. 2005).

In adaptive radiations on continental landmasses, there is also little support for sympatric speciation. In grouse (Tetraoninae), a recently evolved Holarctic subfamily with many widely distributed, sympatric species and numerous adaptive physiological and morphological characters (Johnsgard 1983; Drovetski et al. 2006), peripatric speciation (divergence of a small peripheral population) appears to be the predominant mode of speciation (Drovetski 2003). In songbirds, molecular phylogenetic studies have shown that co-distributed species within Palearctic regions are the result of multiple colonizations of those regions or post vicariant (allopatric) range expansion, rather than the result of in situ (sympatric) speciation (Voelker 1999, 2002; Johannson et al. 2007; Voelker 2010).

The diversity of habitats along environmental gradients is thought to provide an opportunity for sympatric speciation to occur. Mountain areas are good examples of sharp elevational habitat gradients. Furthermore, mountain areas are known hot spots of avian diversity and endemism (Myers et al. 2000; Orme et al. 2005). However, the potential link between speciation across sharp elevational gradients and high avian endemism and diversity is not well supported. Studies in the mountains of South and Central America, hosting the richest avian diversity in the world, show that closely related lineages (e.g., sister taxa) are not found in different habitats along elevational gradients as would be expected under a sympatric speciation model. Instead, they are found in similar environments on different parts of single mountain ranges or on different ranges (Chaves and Smith 2011; Barrera-Guzmán et al. 2012; Gutiérrez-Pinto et al. 2012); these patterns suggest allopatric divergence. A similar pattern occurs in Africa, where studies of a variety of lineages (avian and non-avian) indicate that sister taxa are not co-distributed on single mountains (Voelker et al. 2010). While support for sympatric speciation in birds remains elusive, studies of montane-distributed groups which occupy different habitats may still provide the best opportunity to identify cases of ecological divergence supportive of sympatric speciation.

**Figure 1:**
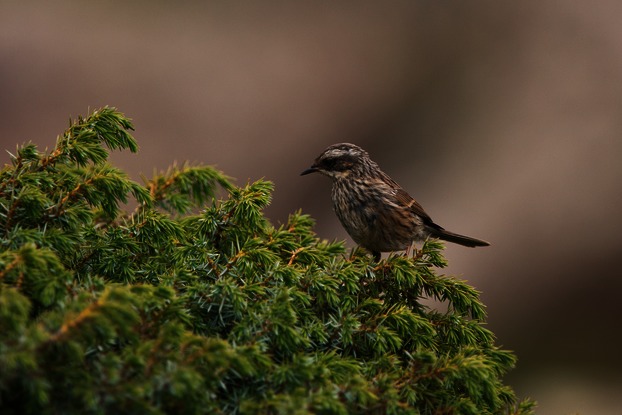
Radde's accentor (*Prunella ocularis*), adult female. Photo taken by S. V. D. on Mt. Aragats, Armenia.

**Figure 2:**
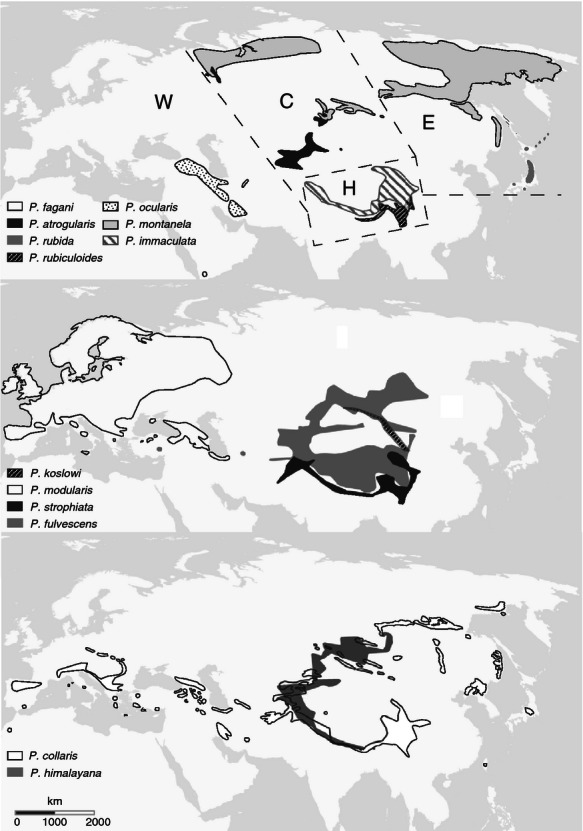
Species ranges of accentors and biogeographic areas (top map) used in our analysis. W, western Palearctic; C, central Palearctic; H, Himalayan region; E, eastern Palearctic.

The Palearctic passerine family, Prunellidae (accentors; Fig. 1), provides such an opportunity. This family consists of a single genus (*Prunella*) comprising 13 species (Dickinson 2003), all of which are associated with mountains and none are long-distance migrants (Hatchwell 2005). Most accentors occur in the Himalayan region, broadly defined to include the Himalayas, Tibet, and mountains of south-central China east of Tibet, or in the central Palearctic (Fig. 2). Three species (*rubiculoides*, *immaculata*, and *strophiata*) are endemic to the Himalayan region, two (*atrogularis* and *koslowi*) are endemic to central Palearctic mountains, and two (*fulvescens* and *himalayana*) are distributed across these two regions (Fig. 2). Three species (*fagani*, *modularis,* and *ocularis*) are restricted to the western Palearctic. One (*rubida*) is endemic to Japan and Kuril islands, *montanella* is distributed across central and eastern Palearctic, and *collaris* is distributed across all regions (Fig. 2).

Importantly for an assessment of possible sympatric speciation in montane systems, habitat associations among accentors vary along an elevational gradient, from high alpine to lower montane forest habitats. Four of the thirteen species (*montanella, koslowi, rubida,* and *modularis*) can also be found in lowlands.

This variation in habitat associations has been used taxonomically, with some authors subdividing *Prunella* into two subgenera or genera: *Laiscopus* and *Prunella* (Stepanyan 2003; Hatchwell 2005). *Laiscopus* includes two larger species (*collaris* and *himalayana*), which breed in alpine habitats, while *Prunella* includes the remaining 11 species, all of which are smaller than *Laiscopus* and associated with shrub habitats inside forest or adjacent habitats. The Himalayas and south-central Siberian regions are each inhabited by both of the *Laiscopus* species and by four *Prunella,* suggesting the possibility that speciation in these regions resulted from ecological divergence (sympatric speciation) rather than geographic isolation (allopatric speciation).

Therefore, in this study we focused on the evolutionary relationships, biogeographic history, and geographic mode of speciation in Prunellidae. We used sequences of the mitochondrial ND2 gene and intron 9 of the Z chromosome specific Aconitase 1 gene (ACO1I9) to reconstruct a phylogeny of all currently recognized species in the family. This phylogeny is then utilized to reconstruct the biogeographic history of Prunellidae and to test whether these mountain specialists diverge in allopatry or in sympatry.

## Material and Methods

### Sampling

We obtained tissue samples of all 13 currently recognized species of Prunellidae (Appendix S1). As outgroups, we used five species of Motacillidae (*Anthus gustavi*, *Motacilla alba*, *M. cinerea*, *M. citreola*, and *M. flava*). We also included one species of Passeridae (*Passer domesticus*), and two species of Ploceidae (*Plocepasser mahali* and *Quelea quelea*). These families appear to be closely related to Prunellidae (Jønsson and Fjeldså 2006; Johansson et al. 2008) and this relationship was supported by our Neighbour-Joining analysis of ND2 sequences of a broad suite of possible passerine outgroups available on GenBank.

### Molecular methods

Genomic DNA was extracted using the JETQUICK Tissue DNA Spin Kit (Genomed, Löhne, Germany) according to the manufacturer's protocol. For all individuals we sequenced the complete mtDNA ND2 gene (1041 bp GenBank accession numbers KC759264 - KC759315, we also used two additional accessions AF407039 and HM538386) and intron 9 of the Z chromosome specific Aconitase 1 gene (ACO1I9, 1094 bp; GenBank accession numbers KC759183 - KC759263). We used previously published polymerase chain reaction (PCR) profiles and primers for both ND2 and ACO1I9 (Drovetski et al. 2004a, 2010). We could not amplify both loci in their entire length for *fagani* (*n* = 2) and *atrogularis* from the Urals (*P. a. atrogularis*; *n* = 2) due to the poor quality of DNA extracted from toe pads of museum specimens. For these species we used five primer pairs for each locus (Appendix S2), but we were still unable to sequence ACO1I9 for the two *atrogularis* from the Urals. We also failed to amplify the 3' half of ACO1I9 for both *collaris* from Sikhote-Alin' mountains (*P. c. erythropygia*), so we used only 544 bp for these samples which we amplified with primers ACO1I-9F and ACO1-I9Rint (Kimball et al. 2009).

PCR fragments were sequenced in both directions at the Macrogen Europe (Netherlands) facility on an ABI 3730 Genetic Analyzer (Applied Biosystems Inc., Foster City, CA). The sequences were aligned automatically in Sequencher 5.0.1 (Gene Codes Corporation, Ann Arbor, MI) and verified manually to ensure consistent alignment of indels in the ACO1I9 sequences.

Females are hemigametic in their Z-specific (sex-linked) loci and thus have only one allele. In heterogametic males whose ACO1I9 alleles differed in length, the alleles were identified by subtracting the complimentary sequence of the allele without the indel from the double peaks in their chromatogram. Although tedious, this procedure allowed us to identify indels and to resolve nucleotide polymorphisms without ambiguity.

ACO1I9 alleles of heterogametic males that had the same length but contained multiple nucleotide differences were resolved using PHASE 2.1.1 (Stephens et al. 2001). We conducted two independent PHASE runs. The first 500 interactions were discarded as burn-in. The following 5000 iterations used a thinning interval of 10. Known haplotypes from females, homozygous males, and males with a single polymorphic site or indel were set as known alleles.

We used *BEAST v1.7.4 (Drummond et al. 2012) to reconstruct phylogenetic trees for each locus and to reconstruct a species tree for Prunellidae using both loci. *BEAST was also used to estimate divergence times across lineages. We used the mean rate of sequence evolution and associated 95% confidence interval (CI) reported by (Lerner et al. 2011) for ND2 (2.9 × 10^−2^ [2.4–3.3 × 10^−2^]). This rate is derived from the sequence of lineage splits in Hawaiian Honeycreepers (Drepanidinae) and is calibrated using the well-established dates of sequential uplift of the Hawaiian Archipelago. For ACO1I9 we allowed the rate to be estimated relative to that of ND2. This estimate was 3.2 × 10^−3^ (95% CI: 2.4–4.0 × 10^−3^). This rate was slightly higher than the evolutionary rates reported for 13 autosomal loci 3.5 × 10^−4^–1.9 × 10^−3^ (Lerner et al. 2011). The higher evolutionary rates for Z-specific as compared to autosomal loci are expected due to the former having ¾ of the population size of the latter. Gene regions were unlinked and allowed to estimate clock parameters independently.

We used the Akaike information criterion (AIC; Akaike 1974) implemented in jModelTest (Posada 2008) to select substitution models for the BEAST analyses. For all loci jModelTest selected slightly simplified submodels of the generalized time reversible (GTR) model (Tavaré 1986) with discrete-gamma (G) model of substitution rates across sites (Yang 1994) and the proportion of invariable sites (I) included. Therefore, we selected GTR + G + I model for BEAST analyses of both loci. We incorporated a Yule process speciation prior for both loci. To select the appropriate molecular clock prior we conducted two independent runs for each locus. In one run we used a strict clock prior and in the other relaxed lognormal prior. We then conducted the maximum likelihood ratio test (MLRT; Huelsenbeck and Crandall 1997) to determine whether the strict clock tree likelihood was significantly worse than the relaxed clock tree likelihood. Because MLRT was not significant for either of our two loci, we used strict molecular clock model in our BEAST analyses.

Three separate MCMC analyses were run for 3 × 10^7^ generations with a 10% burn-in and parameters sampled every 1 × 10^3^ steps. Independent runs were combined using LogCombiner v.1.7.4 (Drummond et al. 2012). Tracer v1.5 (http://beast.bio.ed.ac.uk/Tracer) was used to determine the effective sample size of each parameter and calculate the mean and 95% highest posterior density interval (95% HPD) for divergence times. Tree topologies were assessed using TreeAnnotator v.1.7.4 (Drummond et al. 2012) and visualized in FigTree v.1.3.1 (http://tree.bio.ed.ac.uk/software/figtree/). For individual locus analyses we used all individual ND2 haplotypes and ACO1I9 alleles. For the combined analysis of two loci homozygous males, like females, were represented by a single ACO1I9 + ND2 sequence pair. Heterozygous males were represented by two sequence pairs – the same mtDNA haplotype was used twice to pair it with each of the two different ACO1I9 alleles of that male.

#### Historical biogeography and geographic mode of speciation

Geographic range data were obtained from Atlas der Verbreitung palaearktischer Vögel (http://www.staff.uni-mainz.de/martens/atlas/english.html). For *modularis*, *collaris,* and *montanella*, we used additional maps prepared by Ya. Red'kin, E. Koblik, and A. Mosalov for the new edition of Birds of Russia (unpubl.). These additional maps are based on a large body of Russian literature and locality data associated with specimens housed in several Russian Museums. Species range maps were digitized in MapInfo Professional v. 9.5.1 (Pitney Bowes Inc., Stamford, CT). Although graphic representation of species ranges is inevitably simplistic and not completely accurate, these inaccuracies affect all species to a similar degree and do not bias our results. The digitized species ranges were used to calculate their area, the area of clade ranges, and measure range overlap among clades and species.

We used two approaches to analyze geographic modes of speciation in Prunellidae. One utilizes a node-based approach that compares the ranges of two clades connected by a node on the phylogenetic tree (Barraclough and Vogler 2000). Clade ranges are calculated as joint ranges of clade-member species. These joint ranges are used to calculate the level of sympatry for sister clades (as the sympatry index = range overlap divided by the smaller of the two sister clade ranges). Sympatry values for all nodes are then regressed onto the node ages. If speciation is predominantly allopatric, the most recent divergence events in the phylogeny are expected to have no sympatry. As the ranges change through time, sympatry can only increase. Clustering of 0 sympatry values at younger node ages and presence of non-zero sympatry values at older node ages results in a positive correlation between the node age and sympatry. The opposite relationship is expected for predominantly sympatric speciation (Barraclough and Vogler 2000; Drovetski 2003).

The second approach to determining the geographic mode of speciation is similar, but it utilizes mean and maximum range overlaps among all species in a clade that are regressed onto node age (Fitzpatrick and Turelli 2006). The expectations are similar to the first approach – predominantly allopatric speciation will result in little or no sympatry among species from closely related clades and non-zero sympatry among species from distantly related clades. Predominantly sympatric speciation will result in a negative correlation between the node age and sympatry.

In the case of allopatric speciation, the range symmetry index (the smaller clade range divided by the sum of both clade ranges) can be regressed onto the node age to determine whether the speciation was predominantly peripatric (Barraclough and Vogler 2000). Peripatric speciation is expected to result in low range symmetry for the recent nodes of the phylogenetic tree, but should increase through time because range expansion of a small peripherally isolated clade is more likely to increase with time than decrease. Both sympatry and symmetry indexes are proportions with values bounded by 1 and 0.5, respectively. In order to use them in regression analyses, they were arcsine transformed. The symmetry index values were doubled prior to arcsine transformation.

For our historical biogeography reconstruction we used maximum likelihood analysis of geographic range evolution based on the dispersal-extinction cladogenesis model implemented in LaGrange v. 2.0.1 (Ree and Smith 2008). LaGrange identifies the geographic areas that are included in the most probable ancestral range and which areas were “inherited” by its descendants, for each node along the phylogenetic tree. Using distribution maps, each species was coded as being present or absent in each of four areas: Western, Central and Eastern Palearctic, and the Himalayan region (Fig. 2). Default software assumptions were used. Ancestral areas were reconstructed by performing likelihood optimizations on our species tree.

## Results

### Phylogeny of Prunellidae

Our analysis of individual loci (ND2 and ACO1I9) resulted in similar topologies and time estimates (Appendixes S3, S4, respectively). The differences between these individual loci trees were only in a few nodes that were poorly supported in both topologies. Therefore, we only report results of our species tree reconstruction using both loci.

**Figure 3:**
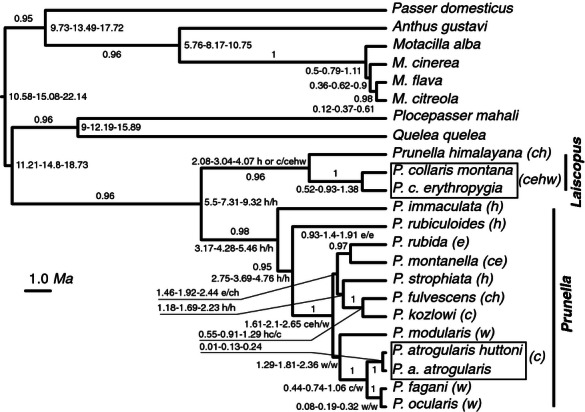
Species tree estimated using ND2 and ACO1I9 sequences. The tree was reconstructed in BEAST v1.7.4 (Drummond et al. 2012) using GTR + G + I model of substitutions, Yule process speciation and strict molecular clock priors for both loci. Substitution rates, models, and topologies were unlinked. The letter(s) in parentheses following taxon names represents the geographic regions (see Fig. 1) that contain the breeding range of the taxon. A single number next to the branch shows its posterior probability, whereas a series of three numbers connected by dashes next to the nodes represent, left to right, the minimum 95% HPD interval, mean, and its maximum 95% HPD interval for the node age in million years. Letters separated by a slash represent geographic areas of occurrence of the node's descended lineages: letter(s) to the left of a slash represents geographic area(s) inherited by the descendent lineage above the node, and letter(s) to the right of the slash represents inherited range of the descendent below the node. GTR, generalized time reversible; HPD, highest posterior density.

The two-loci phylogenetic analysis strongly supported the monophyly of Prunellidae (*PP* = 0.96; Fig. 3). As in the single gene analyses, Prunellidae was divided into two strongly supported clades (both *PP* ≥ 0.96, Fig. 3), one comprising the two large accentors and the other comprising the 11 smaller accentors.

In the clade of smaller accentors, both *immaculata* followed by *rubeculoides* were the most divergent (Fig. 3). However, in contrast to analyses of individual loci these relationships were well supported (*PP* ≥ 0.95). Also in contrast to single loci analyses, the remaining nine small accentors formed two clades. One of these clades consisted primarily of species distributed in the eastern and central Palearctic while the other consisted of western Palearctic species along with a single central Palearctic species (Fig. 3). However, just four of the eight nodes comprising these clades were strongly supported. These strongly supported relationships were sister relationships between *koslowi* and *fulvescens* (*PP* = 0.97), *montanella* and *rubida* (*PP* = 1), and *ocularis* and *fagani* (*PP* = 1). The close relationship of the latter pair to *atrogularis* was also strongly supported (*PP* = 1).

### Historical biogeography and geographic mode of speciation

The family Prunellidae appears to split from outgroup families ∼14.8 Ma (middle Miocene; Fig. 3). The divergence of the large (*Laiscopus)* and small (*Prunella)* accentors occurred 7.31 Ma during the late Miocene. Ancestral area reconstructions indicate a Himalayan region origin for Prunellidae and *Prunella*, and possibly for *Laiscopus* as well (Fig. 3).

The *Laiscopus* accentors *collaris* and *himalayana* diverged from each other 3.04 Ma during the late Pliocene, in either the Himalayan region or the central Palearctic (Fig. 3). The two subspecies of *collaris* (*erythropygia* and *montana*) diverged 0.93 Ma during the early Pleistocene. This divergence predated those between *fulvescens* and *koslowi* (0.91 Ma), *ocularis* and *fagani* (0.19 Ma), and *atrogularis* and the latter pair (0.74 Ma; Fig. 3).

The diversification of small *Prunella* accentors began 4.28 Ma during the early Pliocene when *immaculata* diverged from the common ancestor of other small species (Fig. 3). The second species to diverge within *Prunella* was *rubeculoides*, also during the early Pliocene. Both these divergences happened within the Himalayan region (Fig. 3).

In the early Pleistocene (2.1–1.69 Ma) small accentors experienced a period of rapid radiation that resulted in four divergence events. These divergences are related to dispersal out of the Himalayan region and widespread colonization of the Palearctic (Fig. 3). First, the primarily western Palearctic clade (*modularis*, *atrogularis*, *fagani*, and *ocularis*) diverged from the primarily central and east Palearctic clade (*rubida*, *montanella*, *strophiata*, *fulvescens*, and *koslowi*). This was followed by a divergence of *modularis* from the other primarily west Palearctic accentors, and divergences leading to two sister pairs of central and eastern Palearcic species (*montanella*/*rubida* and *fulvescens*/*koslowi*) and *strophiata* (Fig. 3).

The divergences separating *montanella* from *rubida* (1.4 Ma) and *fulvescens* from *koslowi* (0.91 Ma) happened during the early Pleistocene. The three most recent splits between *atrogularis* and the sister pair of *ocularis* and *fagani* (0.74 Ma), between the latter two species (0.19 Ma), and between the two *atrogularis subspecies* (0.13 Ma) happened during the middle Pleistocene (Fig. 3).

**Figure 4:**
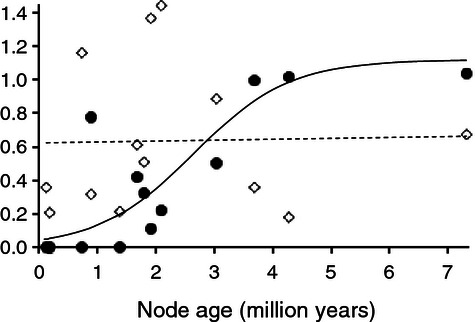
Clade sympatry (black dots, solid line) and clade range symmetry (white diamonds, dashed line) versus node age. Sympatry was arcsine transformed, and symmetry was doubled and arcsine transformed (Barraclough and Vogler 2000).

**Figure 5:**
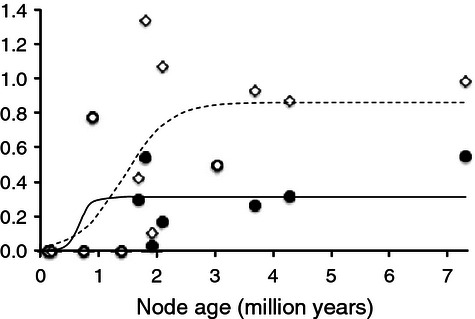
Mean species sympatry (black dots, solid line) and maximum species sympatry (white diamonds, dashed line) versus node age. Values were arcsine transformed, and symmetry was doubled and arcsine transformed (Fitzpatrick and Turelli 2006).

Node sympatry values (Barraclough and Vogler 2000) were positively correlated with node age and the intercept was not significantly different from 0 (node sympatry = 0.019 + 0.170 × Age; *r*² = 0.63, df = 13, *P <* 0.001, intercept *P* = 0.86). However, the logistic sigmoidal curve provided a more appropriate fit to the data (node sympatry = 1.125/(1 + 27.810 × e^−1.185 × Age^); *r*^2^ = 0.71, df = 13, *P <* 0.001; Fig. 4). The best-fit model for both the mean and maximum sympatry among species (Fitzpatrick and Turelli 2006) was also the logistic sigmoidal curve (Fig. 5), although the fit was not as good, especially for the mean species sympatry: max sympatry = 0.861/(1 + 40.405 × e^−2.577 × Age^), *r*^2^ = 0.45; mean sympatry = 0.280/(1 + 4958.433 × e^−11.636 × Age^), *r*^2^ = 0.20. These relationships suggest that clades and taxa within Prunellidae diverge in allopatry.

There was no relationship between clade range symmetry and age (Fig. 4). The range symmetry fluctuated around the mean of 0.368 ± 0.359 throughout the phylogenetic tree of Prunellidae. The lack of the relationship between the range symmetry and age is inconsistent with peripatric speciation.

## Discussion

### Phylogeny of Prunellidae and taxonomic implications

The topologies of our locus specific and combined dataset trees showed a great deal of similarity suggesting an overall lack of conflict between phylogenetic signal in the mitochondrial ND2 gene and the Z chromosome specific ACO1I9. The topologies supported, (1) the monophyly of Prunellidae, (2) two major clades within the family, (3) strongly supported relationships between several species, and (4) strongly supported divergence between subspecies of both *collaris* and *atrogularis* (Fig. 3; Appendixes S3, S4).

The two major clades of Prunellidae separated the larger, alpine species (*collaris* and *himalayana*) from smaller accentors associated with shrubs or scrub habitats (Hatchwell 2005). Due to the level of divergence between these clades, as well as the distinct ecological and morphological differences between them, recognition of *Laiscopus* and *Prunella* as distinct genera may be warranted.

All topologies also suggested that *immaculata* and *rubeculoides* are distantly related to other species in the strongly supported clade of small *Prunella* accentors. The relationship of these two species relative to each other and to remaining species in the clade of small accentors was well supported only in the species tree topology, which suggests that *immaculata* was the first to diverge from the common ancestor of the small accentors, with *rubeculoides* diverging more recently.

Among the remaining species within the clade of smaller accentors not all relationships were strongly supported. The sister relationship of *rubida* and *montanella*, of *fulvescens* and *koslowi*, and of *ocularis* and *fagani* were well supported as were the sister relationships of the subspecies pairs of *collaris* and *atrogularis* and the close relationship of *atrogularis* to *ocularis* + *fagani* (Fig. 3). Deeper divergences in this clade were poorly supported although the combined analysis produced geographically coherent topology eliminating the need to invoke chance long-distance dispersal events. We speculate that the likely explanation for such a lack of resolution is the rapid radiation of these lineages stemming from their colonization of new areas of the Palearctic.

Inclusion of two subspecies of both *collaris* and *atrogularis* into our study revealed inconsistencies of their taxonomic treatment relative to other Prunellids. The divergence between *collaris* subspecies was greater than divergence among *atrogularis*, *fagani*, and *ocularis*, and between *fulvescens* and *koslowi* (Fig. 3). The amount of divergence between these two subspecies suggests the need for a study of genetic variation across the range of *collaris* that could result in elevating some *collaris* subspecies to specific status.

Although the divergence between *atrogularis* subspecies was much lower than the divergence between the two subspecies of *collaris*, it was very similar to the divergence between *fagani* and *ocularis*, which are treated as distinct species. The treatment of the former pair as subspecies and the latter as species may not be justified given that both pairs are allopatric and have equally minor phenotypic differentiation. Given their geographic isolation from one another, and the lineage sorting apparent in our species tree, we suggest that it might be reasonable to elevate these *atrogularis* subspecies to specific status.

### Historical biogeography and geographic mode of speciation

Given the scarce paleorecord for Prunellidae that predates the late Pleistocene (Tyrberg 1998) we used the evolutionary rates reported by (Lerner et al. 2011) for Hawaiian honeycreepers (Fringillidae) to date divergence events in our phylogeny of Prunellidae. However, two records allow us to compare fossil dates with the divergence ages on our species tree. The first fossil date is for *modularis* from Mallorca Is., dated to 1.64–1.80 Ma, and the second is for *collaris* from continental Spain, dated to 0.8 Ma (Tyrberg 1998). In our species tree, the node separating *modularis* from its sister clade is dated to 1.81 (95% HPD 1.29–2.36) Ma, and the split between *collaris* subspecies is dated to 0.93 (95% HPD 0.52–1.38) Ma. Although our molecular divergence dates are slightly older than these paleorecords, the latter are well within the 95% HPD intervals of the former. Furthermore, paleorecords represent minimum ages. Thus, we feel that our molecular dating of the Prunellidae phylogeny is reliable.

Our reconstruction of the biogeographic history of Prunellidae suggests that the origin of family, divergence of the two subgenera (*Laiscopus* and *Prunella*), and initial diversification events within subgenus *Prunella* happened within the Himalayan region between 14.8 Ma and 3.69 Ma (Fig. 3). Subgenus *Prunella* dispersed out of the Himalayan region and across the Palearctic from the mid- to late-Pliocene between 3.69 Ma and 2.1 Ma (Fig. 3). This colonization of the Palearctic was followed by a rapid radiation of accentors suggesting the importance of colonizing new biogeographic regions and vicariant events resulting from Pleistocene glacial retreats in their speciation history. The most recent diversification events in both subgenera, occurred in the early to middle Pleistocene, and happened within or between Palearctic regions (except *strophiata*; Fig. 3).

Regardless of whether we calculated sympatry between sister clades (Barraclough and Vogler 2000) or sympatry among species ranges (Fitzpatrick and Turelli 2006), the relationship between sympatry and divergence age was best described by the logistic sigmoidal curve (Figs. 5). The reason for the nonlinear relationship between sympatry and age is that all clades and taxa that diverged <1.5 Ma are allopatric. The only exception is the sister pair of *koslowi* and *fulvescens* that diverged 0.91 Ma and have a range overlap of 49%. However, while these species have a substantial geographic overlap in range, there is a striking difference in their habitat preferences: *koslowi* lives in thin scrub, semi desert habitat (which is unusual for accentors), whereas *fulvescens* prefers shrubs near timberline in the mountains (which is typical of small accentors; Hatchwell 2005).

The degree of sympatry among clades and taxa rapidly increased between 1.5 Ma and 3 Ma since divergence. Although there are some divergent taxa that currently have nonoverlapping ranges with other species, the node sympatry, and both maximum and mean species sympatry remain relatively high (Figs. 5). This pattern suggests that allopatric speciation was the predominant geographic mode of speciation in Prunellidae.

Despite the similarity of the pattern of relationships between sympatry and age, the clade sympatry fits the logistic sigmoidal curve much better than the maximum and, especially, the mean species sympatry. The use of individual species ranges greatly increases the variance and reduces the goodness of fit due to the stochasticity of individual range sizes and their location. Consider *fagani* whose range does not overlap with any other species because it is located at the far extreme of the family distribution (Fig. 2). Such a range provides no information about the relationship between the sympatry and age because its sympatry value (0) is determined by a historic accident – the probability of colonizing a small, remote habitat island rather than by interactions with other species. However, the presence of this range has a dramatic effect on the mean species sympatry calculation. Furthermore, inconsistencies in the taxonomic treatment of a group can also have a strong effect on calculations of maximum and mean species sympatry. This is because changes in taxonomic treatment (e.g., recognizing divergent *collaris* races as species) will result in changes in the sizes of species ranges and thus the degree of their overlap with other species, as well as in the number of comparison pairs. Therefore, the more species that are recognized and the smaller ranges that they have, the less likely they will overlap, reducing the probability of discovering a significant relationship between species sympatry and age.

By comparison, the use of node ranges eliminates the effects of historic accidents and inconsistent taxonomic treatments because individual ranges of species forming a clade are joined into a single, combined range where each individual species range has little effect on sympatry calculation. To illustrate this, consider the effect of taxonomic treatment on calculation of sympatry between *modularis* and the clade consisting of *atrogularis*, *fagani*, and *ocularis*. If both subspecies of *atrogularis* are treated as distinct taxa, the sympatry between *modularis* and *P. a. atrogularis* is the maximum sympatry for their shared basal node (0.945). The mean sympatry for this node is 0.269, when *P. a. atrogularis* is recognized as distinct. If, despite their allopatric ranges and genetic differentiation, the subspecies of *atrogularis* are treated as a single species, these values change to 0.061 and 0.013, respectively – a drop greater than an order of magnitude. For the clade sympatry calculation, however, the difference in the taxonomic treatment has no effect.

Although peripatric speciation appears to be the most common geographic mode of speciation in animals (Barraclough and Vogler 2000), we found no relationship between clade range symmetry and age as expected for peripatric speciation. In contrast, this relationship was positive and indicative of peripatric speciation in an avian subfamily with a large Palearctic distribution (grouse, Tetraoninae; Drovetski 2003). In grouse, the combination of widely distributed boreal species and their glacial relict sister taxa in southern montane regions result in the positive relationship between age and range symmetry. Regardless of region, all accentor species are closely associated with mountains where habitats are not continuous, but rather have a patchy distribution. The size of the habitat patches available to accentors is determined by the physical and geographic characteristics of each individual mountain range. Therefore, a peripatric mode of speciation may not be a widespread driver of divergences in taxa restricted to habitat islands.

Despite abundant overlap in species distributions suggesting possible speciation in sympatry, our results for Prunellidae are consistent with other studies in identifying allopatric speciation as a primary mode of speciation in Palearctic bird lineages (Voelker 1999; Drovetski 2003; Drovetski et al. 2004b, 2010; Voelker 2010). In comparison with those other studies, one significant difference here is the recent and rapid colonization of Palearctic regions, mostly in the late Pliocene to early Pleistocene. This late colonization of the Palearctic and following rapid succession of divergence events suggest a prompt response to glacial retreat, but not necessarily an effect of glacial expansion and contraction on speciation patterns in Prunellidae.

## Biosketches

Sergei Drovetski is a researcher at the Tromsø University Museum. His professional interests include evolutionary biology, molecular and morphological evolution, genetics/genomics, and ecology of speciation, functional morphology, behavioral ecology, biogeography, phylogeography, and systematics of birds.

Gary Voelker is an Associate Professor of Wildlife and Fisheries Sciences and Curator of Birds at Texas A&M University. His interests include the systematics, speciation, and historical biogeography of widely distributed avian lineages, particularly those of the Old World.

## Supporting Information

**Appendix S1.** Samples used in this study, their collection dates and localities.

**Appendix S2.** Primer pairs used for amplification of ND2 and ACO1I9 from degraded DNA samples.

**Appendix S3.** Phylogenetic tree based on complete sequences of mtDNA ND2 gene. Numbers next to the branches show the posterior probability. The tree was reconstructed in BEAST v1.7.4 (Drummond et al. 2012) using GTR + G + I model of substitutions, Yule process speciation and strict molecular clock priors. A single number next to the branch shows its posterior probability, whereas a series of three numbers connected by dashes next to the nodes represent, left to right, the minimum 95% HPD interval, mean, and its maximum 95% HPD interval for the node age in million years.

**Appendix S4.** Phylogenetic tree based on complete sequences of ACO1I9. Numbers next to the branches show the posterior probability. The tree was reconstructed in BEAST v1.7.4 (Drummond et al. 2012) using GTR + G + I model of substitutions, Yule process speciation and strict molecular clock priors. A single number next to the branch shows its posterior probability, whereas a series of three numbers connected by dashes next to the nodes represent, left to right, the minimum 95% HPD interval, mean, and its maximum 95% HPD interval for the node age in million years.
