# Supplementary material for: Geographic mode of speciation in a mountain specialist Avian family endemic to the Palearctic
Source: Ecol Evol. 2013 Apr 18;3(6):1518–28. doi: 10.1002/ece3.539 (PMC3686188; doi:10.1002/ece3.539)
Supplement: Supplementary file 1 [file ece30003-1518-SD1.pdf]

Appendix S1 Samples used in this study, their collection dates and localities.

ID	Institution/Person	Species	Sex	Date	Lat	Lon	Country
SVD4405	SDM	*Anthus gustavi*	F	3-Jun-09	52.81	156.43	Russia
SGA0811	MSUZM	*Motacilla alba*	F		53.92	102.05	Russia
IVF0746	SDM	*Motacilla cinerea*	F	26-Jun-06	44.04	40.85	Russia
BKS1748	UWBM 49351	*Motacilla citreola*	F	3-Jun-94	51.58	37.21	Russia
SVD4468	SDM	*Motacilla flava*	F	10-Jun-09	52.81	156.42	Russia
C063701	CIBIO	*Passer domesticus*	M	4-Aug-09	41.31	-8.73	Portugal
E21340	CIBIO	*Passer domesticus*	F	19-May-10	31.72	-6.97	Morocco
GAV3349	TCWC	*Plocepasser mahali*	?	29-Dec-10	-28.37	24.48	South Africa
GAV0210	UWBM 46587	*Prunella atrogularis*	M	9-Jun-93	51.03	85.65	Russia
R118888	MSUZM	*Prunella atrogularis*	F	22-Sep-36	62.47	59.03	Russia
R118889	MSUZM	*Prunella atrogularis*	M	16-Sep-36	62.47	59.03	Russia
SGA1414	MSUZM	*Prunella atrogularis*	M	19-Jun-11	51.04	85.64	Russia
SGA1419	MSUZM	*Prunella atrogularis*	M	20-Jun-11	51.04	85.64	Russia
EAK64	MSUZM	*Prunella collaris*	M	8-May-04	43.45	41.78	Russia
IVF1091	SDM	*Prunella collaris*	F	13-Jun-11	40.47	44.19	Armenia
MSUZM72000	MSUZM7/2000	*Prunella collaris*	F	27-Jan-98	43.72	42.65	Russia
N6	MSUZM	*Prunella collaris*	F	27-Jun-07	47.11	136.54	Russia
RYA2451	MSUZM	*Prunella collaris*	F	27-Jun-07	47.11	136.54	Russia
SVD2079	UWBM 64765	*Prunella collaris*	M	8-Jul-99	43.82	40.72	Russia
10598	BMNH 1965.M.10598	*Prunella fagani*	M	27-Dec-48			Yemen
10600	BMNH 1965.M.10600	*Prunella fagani*	M	27-Dec-48			Yemen
DAB0245	UWBM 46404	*Prunella fulvescens*	M	25-May-93	42.98	75.88	Kazakhstan
DAB2267	UWBM 57989	*Prunella fulvescens*	F	5-Jun-97	44.90	100.57	Mongolia
DAB0264	UWBM 46423	*Prunella himalayana*	F	27-May-93	42.98	75.88	Kazakhstan
SVD2275	UWBM 66677	*Prunella himalayana*	F	23-Jun-00	50.13	90.05	Russia
15218	KU (Tissue # 15218)	*Prunella immaculata*	?				Myanmar
15219	KU (Tissue # 15219)	*Prunella immaculata*	?				Myanmar
PAH710	KU 114405 (Tissue # 20366)	*Prunella kozlowi*	M	10-Jun-09	43.50	104.01	Mongolia

Appendix S1 (continued) Samples used in this study, their collection dates and localities.

ID	Institution/Person	Species	Sex	Date	Lat	Lon	Country
PAH716	KU 117329 (Tissue # 20375)	*Prunella kozlowi*	M	12-Jun-09			Mongolia
PAH717	KU 114404 (Tissue # 20376)	*Prunella kozlowi*	M	12-Jun-09	43.49	104.07	Mongolia
HLO074	KU 114385 (Tissue # 20401)	*Prunella kozlowi*	M	14-Jun-09	43.49	104.09	Mongolia
PAH762	KU 114406 (Tissue # 20463)	*Prunella kozlowi*	F	1-Jul-09	43.62	103.75	Mongolia
PAH781	KU 115098 (Tissue # 20491)	*Prunella kozlowi*	?	7-Jul-09	43.83	103.29	Mongolia
SGA0666	MSUZM	*Prunella kozlowi*	F	31-May-10	50.02	95.07	Russia
B1331	MCCI-B-1331	*Prunella modularis*	?	pullus	44.97	6.93	Italy
BKS7036	USNM 637507	*Prunella modularis*	F	31-May-06	41.48	24.33	Greece
CBH0877	MSUZM	*Prunella modularis*	F	14-Sep-04	58.39	50.44	Russia
GVA021	MSUZM	*Prunella modularis*	M	5-May-06	54.90	20.28	Russia
KDV05	MSUZM	*Prunella modularis*	F	23-Apr-04	54.84	37.71	Russia
MR0354	MNHB	*Prunella modularis*	F	19-May-08	42.88	19.38	Serbia
SVD4706	MSUZM	*Prunella modularis*	?	12-Aug-11	60.79	59.55	Russia
CSW4550	UWBM 44004	*Prunella montanella*	F	17-Jul-92	60.09	150.79	Russia
SVD0267	UWBM 47361	*Prunella montanella*	M	25-Jun-93	50.77	134.75	Russia
IVF1092	SDM	*Prunella ocularis*	M	13-Jun-11	40.41	44.25	Armenia
IVF1093	SDM	*Prunella ocularis*	M	13-Jun-11	40.41	44.25	Armenia
IVF1094	SDM	*Prunella ocularis*	M	13-Jun-11	40.41	44.25	Armenia
IVF1095	SDM	*Prunella ocularis*	M	13-Jun-11	40.41	44.25	Armenia
IVF1096	SDM	*Prunella ocularis*	M	13-Jun-11	40.41	44.25	Armenia
IVF1101	SDM	*Prunella ocularis*	M	16-Jun-11	40.41	44.25	Armenia
MAR3636	J. Martens	*Prunella rubiculoides*	M	31-Aug-02	35.40	99.43	China
MAR1794	J. Martens	*Prunella rubiculoides*	M	27-Jun-01	36.73	99.78	China
RYA1708	MSUZM R123605/UWBM 45240	*Prunella rubida*	M	14-Jul-04	45.06	147.79	Russia
15220	KU (Tissue # 15220)	*Prunella strophiata*	?				Myanmar
FBG2139	KU 117268 (Tissue # 25162)	*Prunella strophiata*	M	20-Oct-89	33.07	102.80	China
GAV3216	TCWC	*Quelea quelea*	M	26-May-09	-28.37	24.48	South Africa
