# Supplementary material for: Geographic mode of speciation in a mountain specialist Avian family endemic to the Palearctic
Source: Ecol Evol. 2013 Apr 18;3(6):1518–28. doi: 10.1002/ece3.539 (PMC3686188; doi:10.1002/ece3.539)
Supplement: Supplementary file 2 [file ece30003-1518-SD2.pdf]

**Appendix S2** Primer pairs used for amplification of ND2 and ACO1I9 from degraded samples

| | | | |
|---|---|---|---|
| ND2 | | | |
| L5215 | TATCGGGCCCATACCCCGAAAAT | H257 | ATGGCACACAGGACAATGGGA |
| L243 | TCAGCTCTGGTCCTATTCTCCA | H491 | CATAGCCATCCTTTCGGCAG |
| L491 | TAACCCCACCCTCCTAACTACC | H713 | AATGACTGCATGGACAAAGACC |
| L667 | AACTGCGGCTGTATTTCTCACC | H850 | CCCCAGCAGCCACAATCATT |
| L871 | TCCTCCCCAAATGACTAATCA | H1064 | CTTTGAAGGCCTTCGGTTTA |
| ACO1I9 | | | |
| ACO1I9F | CTGTGGGAATGCTGAGAGATTT | ACO1I9R247 | TTAGTTCGATAGGGCTTTGA |
| ACO1I9F251 | GCTGGTACAGATTCTTCAGTTAGT | ACO1I9R520 | GTAACTTGTGTTTGGGTCTGC |
| ACO1I9F486 | CTGTATCCAGGAAAGTCAAGAATA | ACO1I9R698 | GCATGAAGCCATCTTTTAGA |
| ACO1I9F674 | CCAGTATTTCTTCACATCATTTTT | ACO1I9R861 | TTTGATAGCCATACCTGTGAGC |
| ACO1I9F856 | CATTGGGTTTTTCAGTAACTTTAG | ACO1I9R | CTGCAGCAAGGCACAACAGT |
| ACO1I9FintPr | CCTCTGTGGTAACCTCAGAGCA | ACO1I9Rint | TTGTAACTTGTGTTTGGGTCTGC |
