# Supplementary material for: Geographic mode of speciation in a mountain specialist Avian family endemic to the Palearctic
Source: Ecol Evol. 2013 Apr 18;3(6):1518–28. doi: 10.1002/ece3.539 (PMC3686188; doi:10.1002/ece3.539)
Supplement: Supplementary file 4 [file ece30003-1518-SD4.pdf]

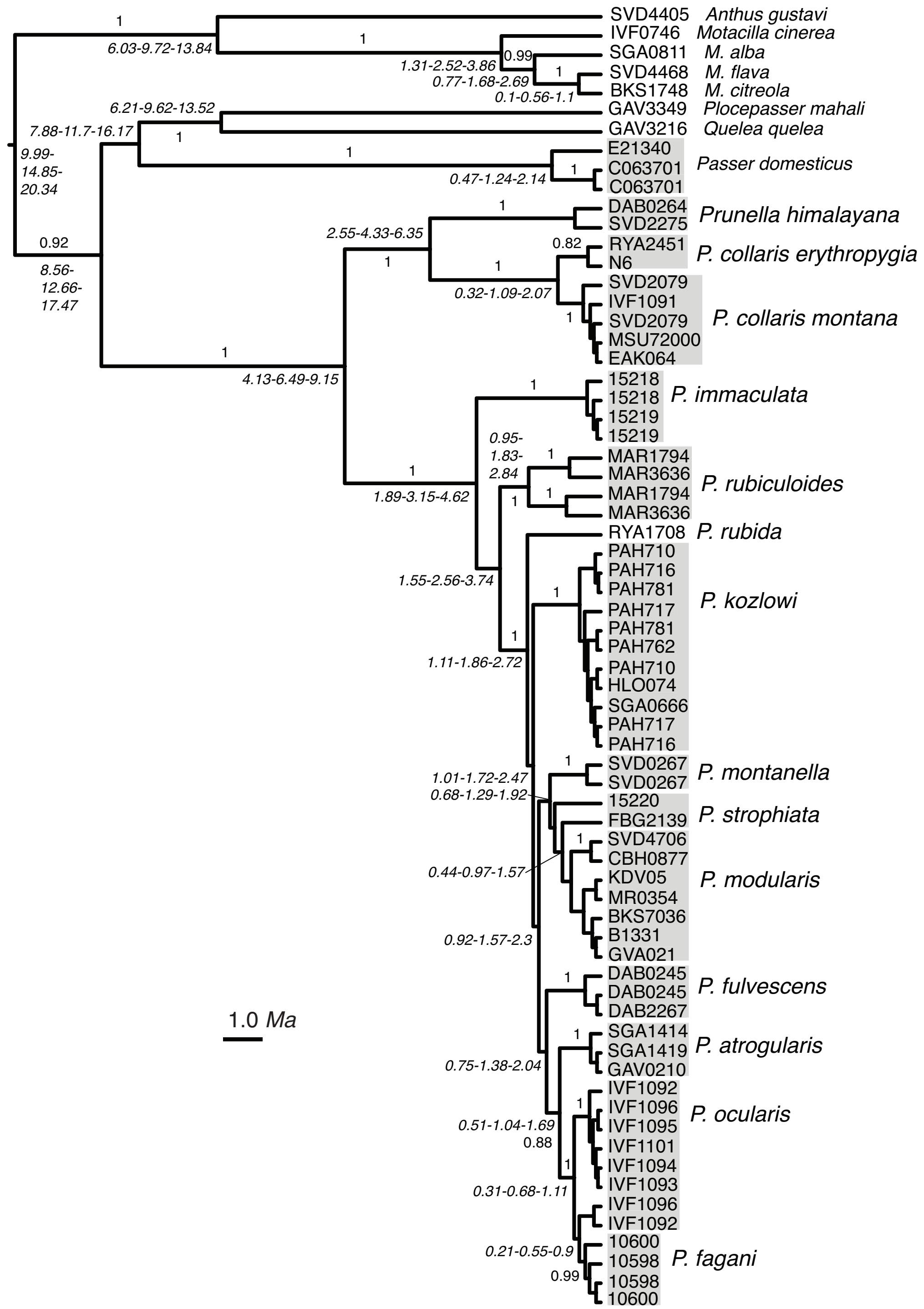

Supplimentary Appendix 4. Phylogenetic tree based on complete sequences of ACO1I9. Numbers next to the branches show the posterior probability. The tree was reconstructed in BEAST v1.7.4 (Drummond *et al.*, 2012) using GTR+G+I model of substitutions, Yule process speciation and strict molecular clock priors. A single number next to the branch shows its posterior probability, whereas a series of three numbers connected by dashes next to the nodes represent, left to right, the minimum 95% HPD interval, mean, and its maximum 95% HPD interval for the node age in million years.
